# Correlation between vitamin D level and severity of prognostic markers in Egyptian COVID-19 patients: a cohort study

**DOI:** 10.1186/s43162-022-00131-x

**Published:** 2022-06-22

**Authors:** Hala Ramadan, Ahmed Mohammed Shennawy

**Affiliations:** Internal Medicine Department, Faculty of Medicine, Cairo University, Cairo, Egypt

## Abstract

The outbreak of coronavirus disease 2019 (COVID-19), which is caused by the highly contagious severe acute respiratory syndrome coronavirus 2 (SARS-CoV-2), was announced a pandemic in March 2020 by the World Health Organization. The disease can be diagnosed on the basis of clinical symptoms, polymerase chain reaction positivity, and the presence of ground-glass opacities on computed tomography (CT) scans.

Recent studies have focused on the role of serum inflammatory markers that predict COVID-19, such as lymphocyte counts and C-reactive protein (CRP), homocysteine, and D-dimer levels. Vitamin D is thought to reduce the risk of viral infections through several mechanisms.

Our aim was to evaluate the correlation between serum vitamin D level and inflammatory markers and severity in Egyptian patients with COVID-19 infection. Serum vitamin D level had a positive correlation with hemoglobin level and lymphocytes.

As results, serum vitamin D had a negative correlation with serum ferritin, CRP, and D-dimer and was not correlated with CORAD scoring in the CT chest.

In conclusion, serum vitamin D was inversely correlated with inflammatory markers (ferritin, CRP, and D-dimer) which mean that participants with symptoms of COVID-19 had a high level of inflammatory markers and a low level of vitamin D.

Participants without symptoms of COVID-19 had normal inflammatory markers and normal vitamin D level.

## Introduction

The outbreak of coronavirus disease 2019 (COVID-19), which is caused by the highly contagious severe acute respiratory syndrome coronavirus 2 (SARS-CoV-2), was announced a pandemic in March 2020 by the World Health Organization. The disease mainly affects the respiratory system and spreads via aerosols released during sneezing and coughing [1, 2].

The main symptoms of COVID-19 are fever, cough, runny nose, nasal congestion, shortness of breath, headache, and myalgia [2, 3]. The disease can be diagnosed on the basis of clinical symptoms, polymerase chain reaction positivity, and the presence of ground-glass opacities on computed tomography (CT) scans [4].

Recent studies have focused on the role of serum inflammatory markers that predict COVID-19, such as lymphocyte counts and C-reactive protein (CRP), homocysteine, and D-dimer levels [5, 6].

The levels of ferritin, a crucial immune response mediator, increase in severe COVID-19 cases. Increased ferritin levels could cause a cytokine storm by exerting direct immunosuppressive and pro-inflammatory effects. D-dimer is a fibrin degradation product used to exclude the diagnosis of thrombosis [7].

Vitamin D, which affects the nuclear vitamin D receptor, enhances innate cellular immunity by inducing antimicrobial peptides. Vitamin D is thought to reduce the risk of viral infections through several mechanisms, and decreased vitamin D levels have been observed in patients with viral pneumonia [8].

Observational studies suggest that vitamin D has some antiviral properties, and vitamin D supplementation might decrease the risk of respiratory infections. These findings are simple but may play a significant role in our attempts to fight against the COVID-19 pandemic and other infections by minimizing health-related complications through simple intervention [9].

### Aim of the work

Our aim is to evaluate the correlation between serum vitamin D level and inflammatory markers and the severity of symptoms in Egyptian patients with COVID-19 infection.

## Patients and methods

Our work was conducted on COVID-19 Egyptian patients non-symptomatic and symptomatic (with mild, moderate, and severe symptoms) in outpatient clinics, Internal Medicine Hospital, Kasr Alainy Hospital, Faculty of Medicine, Cairo University.

### Patient population

1. The symptomatic group (1) with COVID-19 infection included 71 patients who had mild, moderate, and severe symptoms with a positive nasal swab (PCR) for COVID-19 infection and ground-glass opacities (GGO) in computed tomography (CT) chest.
2. The non-symptomatic group (2) included 74 patients who had a positive nasal swab (PCR) for COVID-19 infection but without symptoms.

COVID-19 infection was confirmed by doing a nasal swab (PCR for coronavirus), and a CT chest was done for symptomatic patients.

We measured the following:

- Complete blood count, serum vitamin (D), D-dimer, CRP, and serum ferritin
- Complete blood count was estimated using a cell counter by cell Dyn machine

1. Hb level: male: 13.2–16.6 g/L, female: 11.6–15 g/L
2. TLC: 3400–9600 cells/mcL
3. Platelets: 150,000–450,000 cells/mcL

- Vitamin D was measured by competitive binding methods, high-performance liquid chromatography (HPLC), and radioimmunoassay (RIA)
- Normal vitamin D level: .30–50 ng/ml
- The Alere Triage® D-Dimer Test was used to measure D-dimer in EDTA anticoagulated whole blood and plasma specimens.
- Normal D-dimer level: ˂ 0.05 μg/ml
- CRP was estimated by using nephelometry DN100
- Normal CRP level: ˂ 5 mg/L
- Serum ferritin was measured in a Cobas e601 device with ECLIA being also measured in an immunoturbidimetric Cobas c501 device.
- Normal serum ferritin level: 20–250 ng/ml

- The severity of COVID-19 was assessed by using the modified National Early Warning Score (NEWS) which includes the following points: age, respiratory rate, O2 saturation, systolic blood pressure, heart rate, any O2 supplementation, conscious level, and temperature [10].
- Each item took a score from 0 to 3
- The severity of COVID-19 was classified according to the score from 0 to ≥ 7:
  - 0 score = no risk
  - 1–4 score = low risk
  - 5–6 score = moderate risk
  - ≥ 7 score = high risk

### Inclusion criteria

- COVID-19 Egyptian patients (males and females) ≥18 years old, asymptomatic and symptomatic (with mild, moderate, and severe symptoms)

### Exclusion criteria

1. Patients with active inflammation
2. Patients with autoimmune diseases
3. Patients with recent thrombosis
4. Patients with a history of bone diseases
5. Menopausal women

## Results

Our work was conducted on patients who visited outpatient clinics and patients admitted to the isolation department in Internal Medicine Hospital, Kasr Alainy, Faculty of Medicine, Cairo University.

Patients were divided into two groups:

1. Symptomatic group (71 patients)
2. Non-symptomatic group (74 patients)

All patients (symptomatic and non-symptomatic) had a positive nasal swab for PCR of COVID-19 infection.

### Comparison between groups

#### Data analysis

**Fig. 1:**
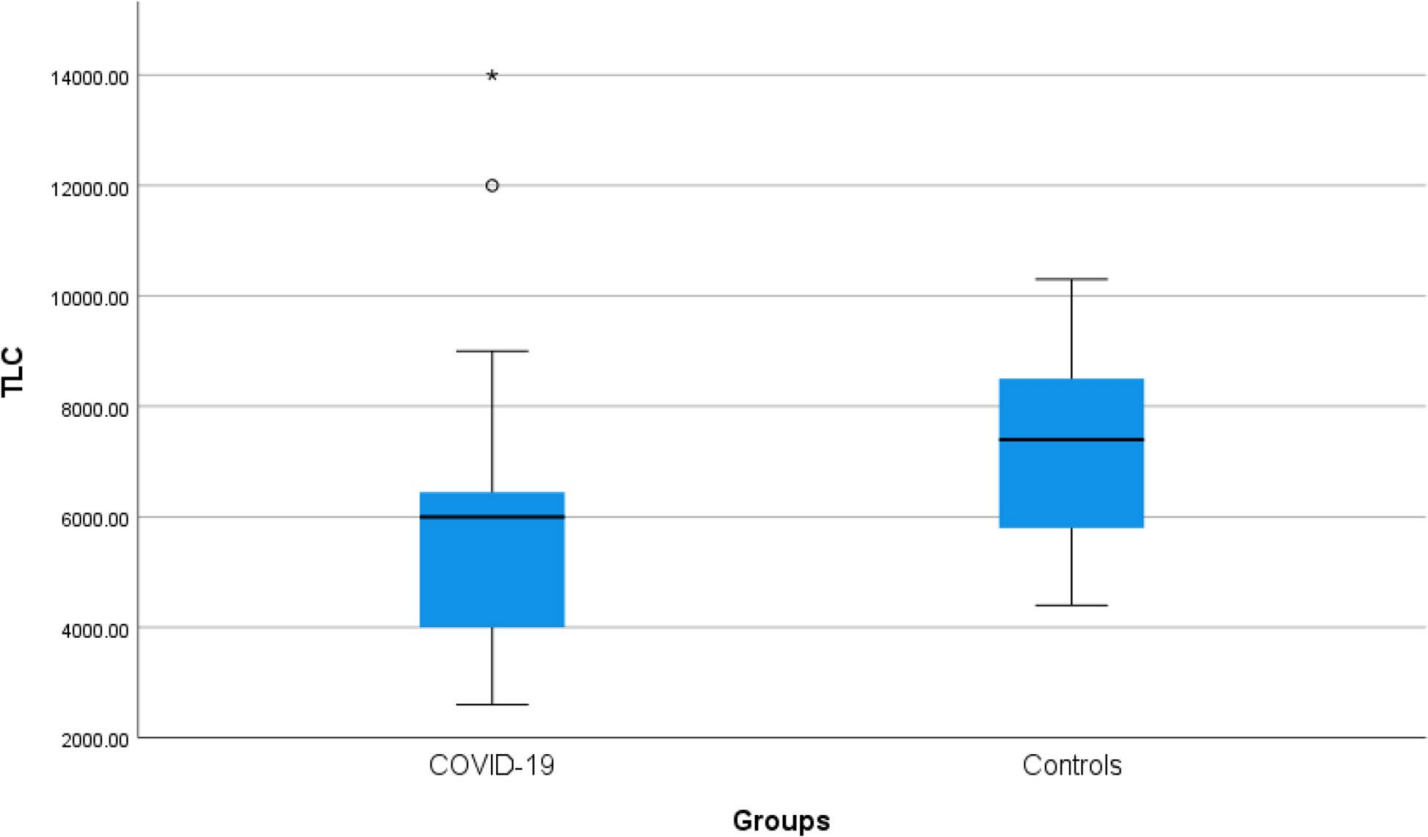
Age of the patients

Non-symptomatic patients were younger than symptomatic patients (*p* value < 0.012) as shown in Table 1 (Fig. 1).

**Table 1:** Age and investigations of the patients

	Symptomatic patients					Non-symptomatic patients					***p*** value
	Mean	SD	Median	Minimum	Maximum	Mean	SD	Median	Minimum	Maximum	
**Age**	32.07	9.97	30.00	18.00	54.00	35.50	5.37	34.50	25.00	49.00	0.012
**SO2 %**	97.58	1.75	98.00	90.00	99.00	97.57	1.09	98.00	95.00	99.00	0.968
**Hb**	12.29	1.46	12.30	9.00	16.00	12.37	0.75	12.20	11.00	14.00	0.685
**PLT**	305.61	87.99	300.00	150.00	560.00	262.32	49.33	261.50	170.00	393.00	<0.001
**TLC**	5526.76	1962.87	6000.00	2600.00	14000.00	7224.32	1649.31	7400.00	4400.00	10300.00	<0.001
**Lymph**	1510.56	543.60	1400.00	200.00	3000.00	3192.57	660.51	3300.00	1800.00	4300.00	<0.001
**Neutro**	4097.62	2423.94	4000.00	300.00	12000.00	3270.27	1053.79	3000.00	1800.00	7500.00	0.082
**Ferritin**	162.69	180.69	87.00	5.00	900.00	53.39	27.18	48.50	17.00	102.00	<0.001
**CRP**	19.53	27.62	11.00	1.00	150.00	2.78	1.21	3.00	1.00	5.00	<0.001
**D-dimer**	0.42	0.24	0.30	0.10	1.20	0.32	0.16	0.30	0.10	0.60	0.023
**Vit D**	14.93	9.25	12.00	2.00	51.00	39.46	4.54	39.00	30.00	49.00	<0.001

**Fig. 2:**
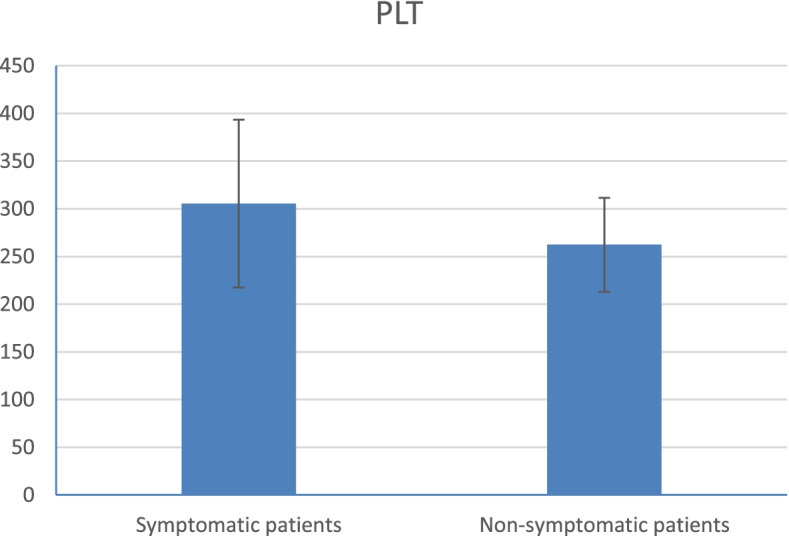
Platelet count of the patients

**Fig. 3:**
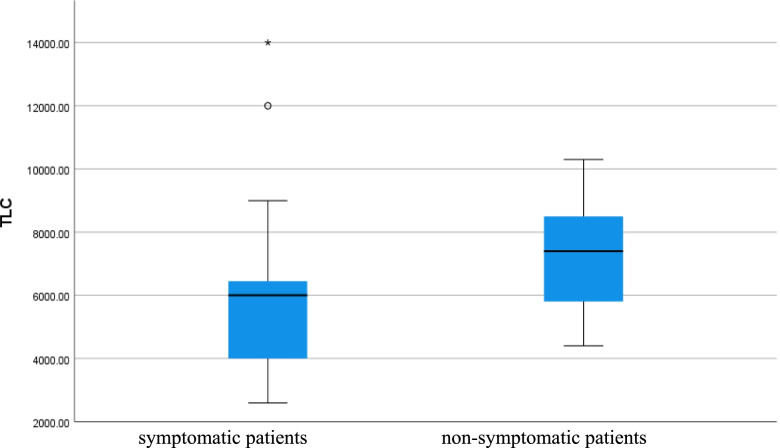
Total leucocytic count of COVID 19 patients (symptomatic and non-symptomatic)

Platelet count and total leucocytic count were higher in symptomatic patients than in non-symptomatic patients (*p* value < 0.001) as shown in Table 1 (Figs. 2 and 3).

**Fig. 4:**
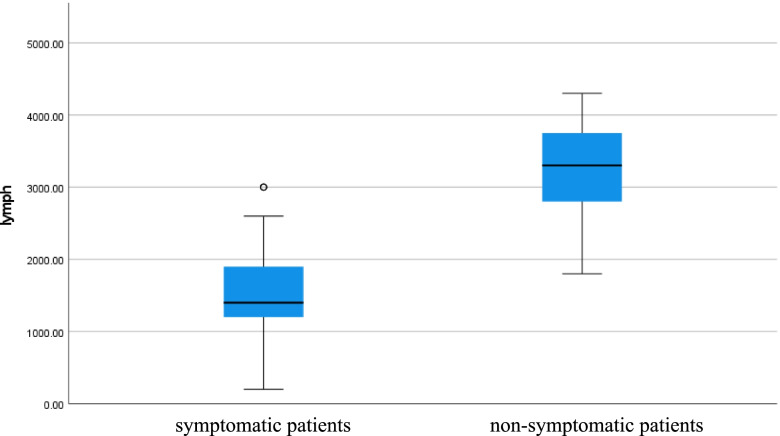
Lymphocytic count of COVID 19 patients (symptomatic and non-symptomatic)

- Lymphocytic count was low in symptomatic patients but normal in non-symptomatic patients (*p* value < 0.001) as shown in Table 1 (Fig. 4).

**Fig. 5:**
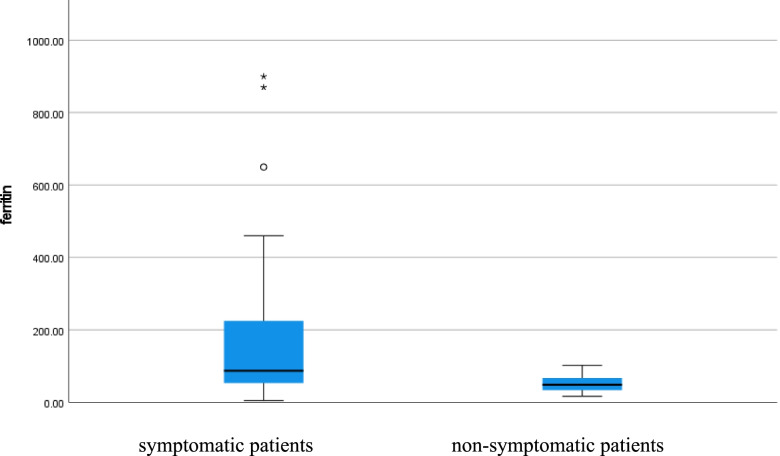
Serum ferritin level of COVID 19 patients (symptomatic and non-symptomatic)

**Fig. 6:**
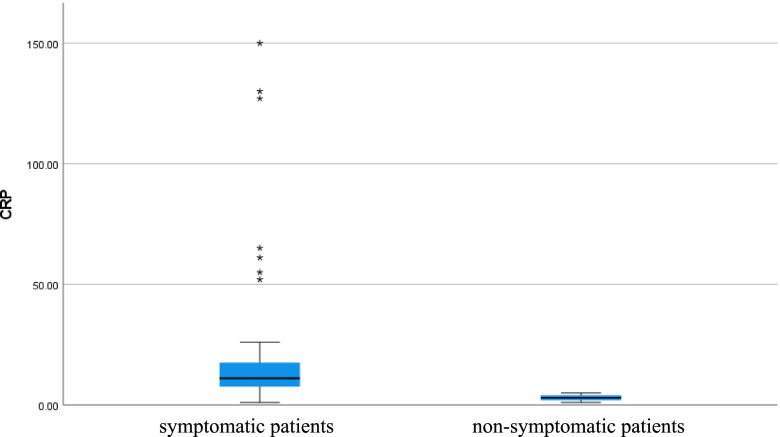
C-Reactive protein level of COVID 19 patients (symptomatic and non-symptomatic)

**Fig. 7:**
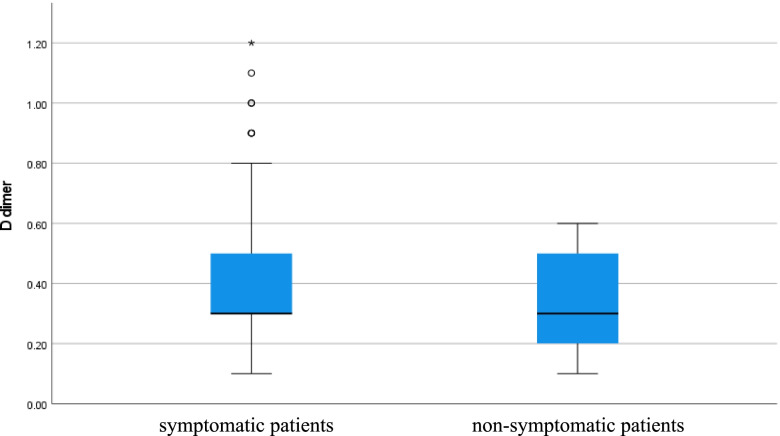
Serum D-dimer level of COVID 19 patients (symptomatic and n0n-symptomatic)

**Fig. 8:**
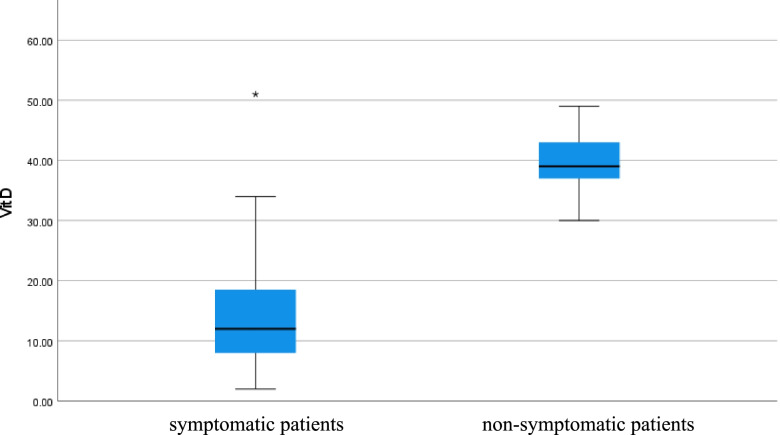
Serum vitamin D level of COVID 19 patients (symptomatic and n0n-symptomatic)

Serum ferritin level was high in symptomatic patients but normal in non-symptomatic patients (*p* value < 0.001), CRP level was high in symptomatic patients but normal in non-symptomatic patients (*p* value < 0.001), D-dimer level was high in symptomatic patients but normal in non-symptomatic patients (*p* value 0.023), and vitamin D level was low in symptomatic patients but normal in non-symptomatic patients (*p* value < 0.001) as shown in Table 1 (Figs. 5, 6, 7, and 8).

According to the severity score of symptomatic patients, the results were that:

1. There were no severe risky patients.
2. There were 21 moderate risky patients.
3. There were 50 mild risky patients (*p* value 0.025) as shown in Table 2.

**Table 2:** Symptoms and comorbidities of the patients

		Symptomatic patients		Non-symptomatic patients		***p*** value
		Count	%	Count	%	
**Gender**	**Male**	19	26.8%	30	40.5%	0.079
	**Female**	52	73.2%	44	59.5%	
**Smoking**	**Yes**	21	29.6%	7	9.5%	< 0.001
	**No**	50	70.4%	67	90.5%	
**DM**	**Yes**	7	9.9%	0	0.0%	0.006
	**No**	64	90.1%	74	100.0%	
**Obesity**	**Yes**	21	29.6%	8	10.8%	0.006
	**No**	50	70.4%	66	89.2%	
**HTN**	**Yes**	8	11.3%	0	0.0%	0.003
	**No**	63	88.7%	74	100.0%	
**Fever**	**Yes**	48	67.6%	0	0.0%	< 0.001
	**No**	23	32.4%	74	100.0%	
**Cough**	**Yes**	44	62.0%	0	0.0%	< 0.001
	**No**	27	38.0%	74	100.0%	
**Anosmia**	**Yes**	30	42.3%	0	0.0%	< 0.001
	**No**	41	57.7%	74	100.0%	
**Diarrhea**	**Yes**	14	19.7%	0	0.0%	< 0.001
	**No**	57	80.3%	74	100.0%	
**CT (CORADS)**	**5**	3	4.2%	0	0.0%	< 0.001
	**4**	2	2.8%	0	0.0%	
	**3**	7	9.9%	0	0.0%	
	**2**	6	8.5%	0	0.0%	
	**1**	53	74.6%	0	0.0%	
	**0**	0	0.0%	74	100.0%	
**Severity**	**Moderate risk**	21	29.6%	0	0.0%	0.025
	**Low risk**	50	70.4%	0	0.0%	

**Fig. 9:**
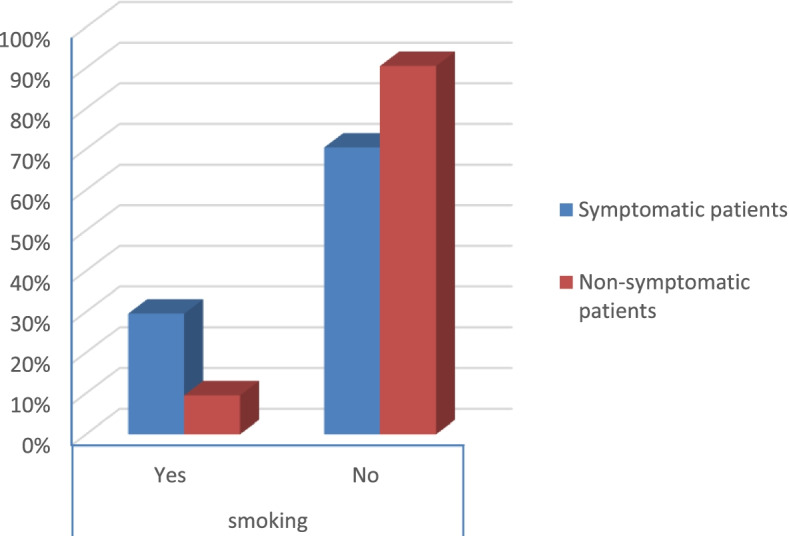
Percentage of smoking in the patients

**Fig. 10:**
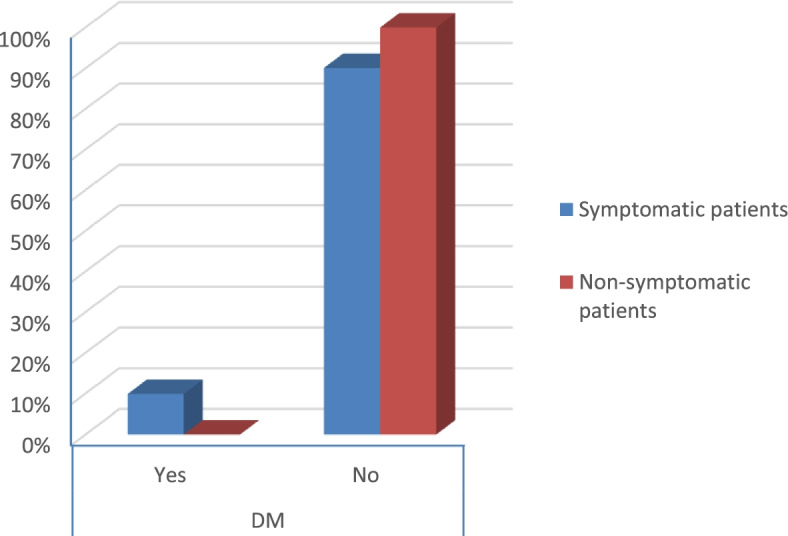
Percentage of DM in the patients

The percentage of smoking and obesity was higher in symptomatic patients than in non-symptomatic patients (*p* value < 0.001 and 0.006) respectively as shown in Table 2 (Figs. 9 and 10).

**Fig. 11:**
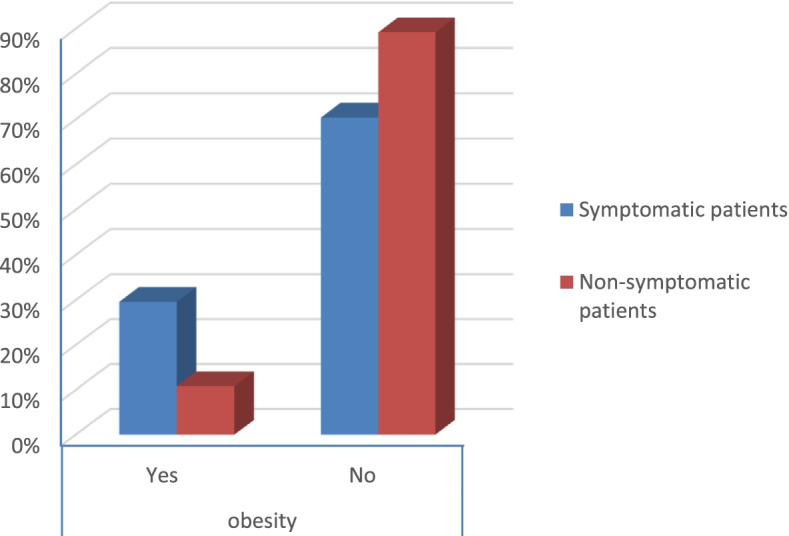
Percentage of obesity in the patients

**Fig. 12:**
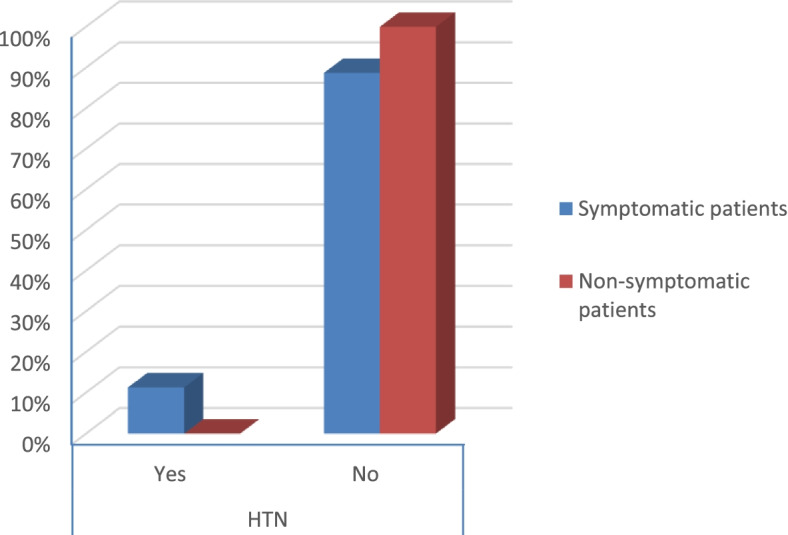
Percentage of HTN in the patients

The percentage of diabetes and hypertension was higher in symptomatic patients than in non-symptomatic patients (*p* value < 0.006 and 0.003) respectively as shown in Table 2 (Figs. 11 and 12).

**Fig. 13:**
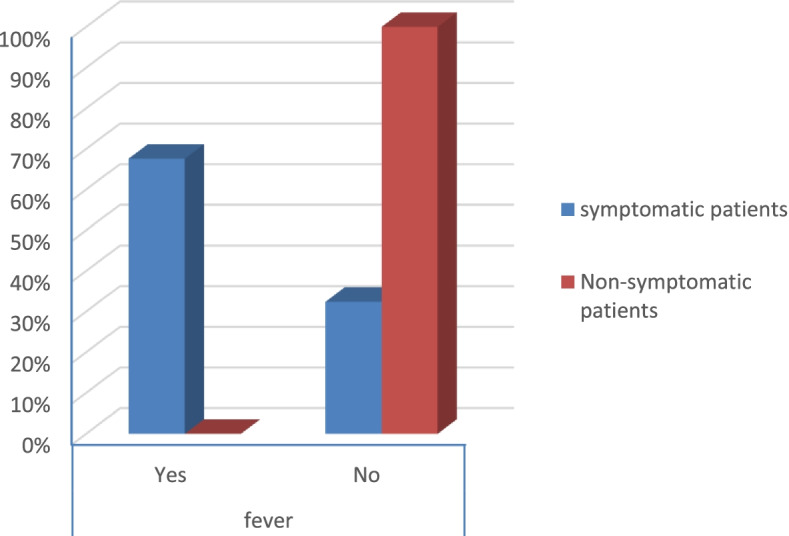
Percentage of fever in the patients

**Fig. 14:**
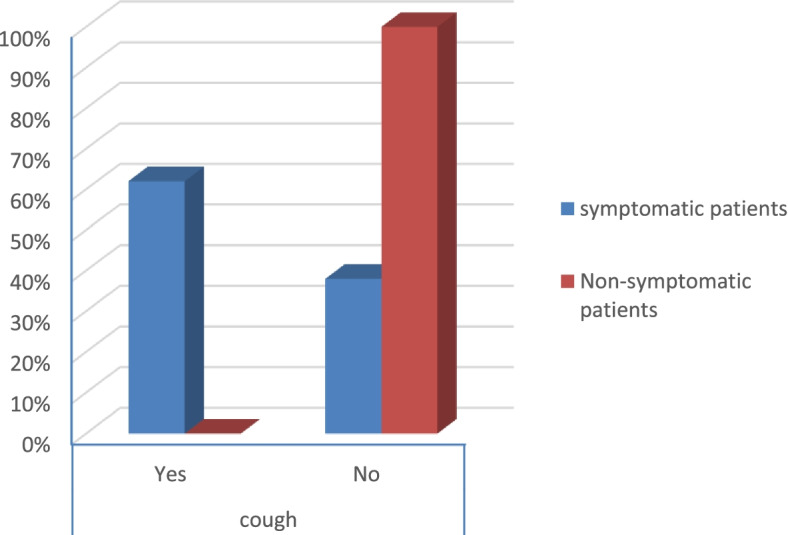
Percentage of cough in the patients

**Fig. 15:**
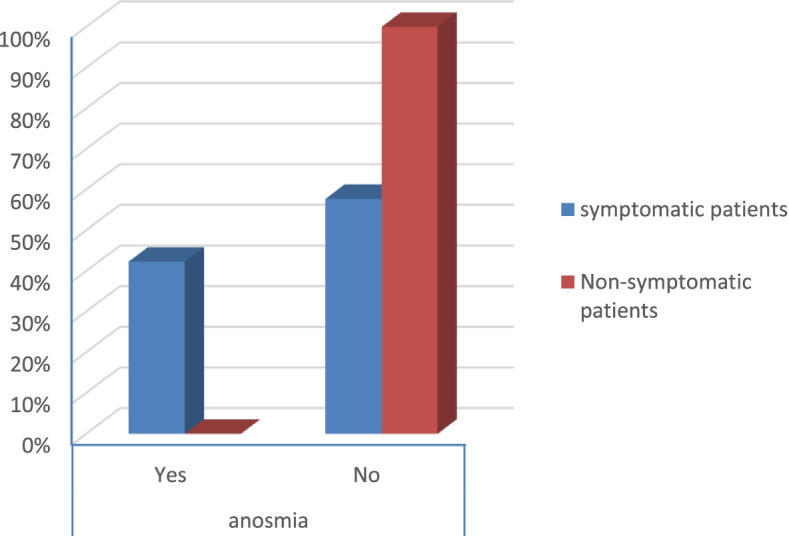
Percentage of anosmia in the patients

**Fig. 16:**
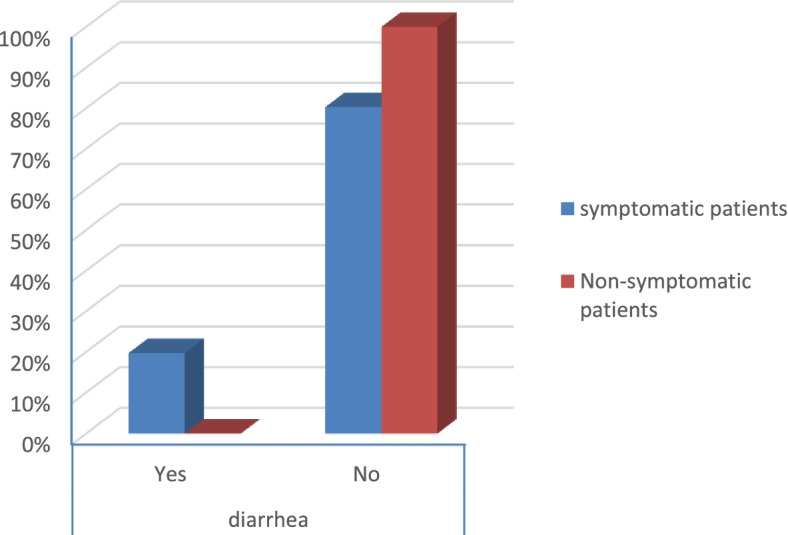
Percentage of diarrhea in the patients

Symptomatic patients presented mostly by fever, anosmia, cough, and diarrhea (*p* value < 0.001) as shown in Table 2 (Figs. 13, 14, 15, and 16).

**Fig. 17:**
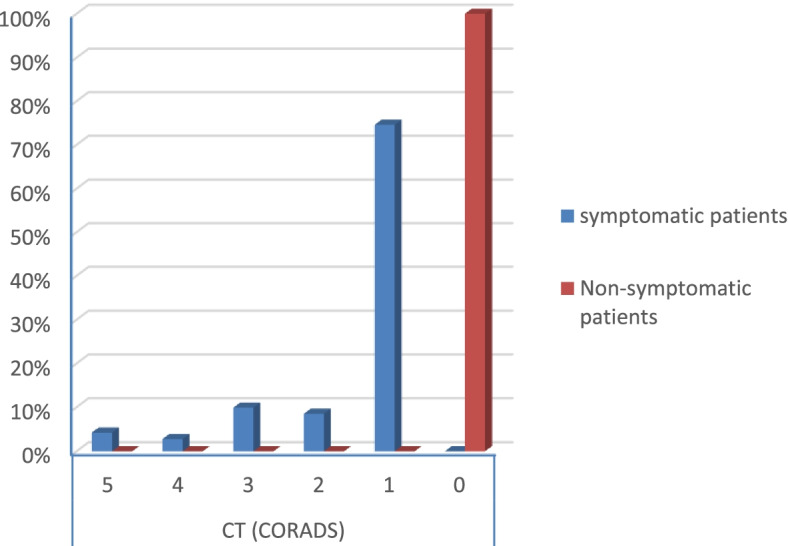
Percentage of CT (CORADS) staging in the patients

Symptomatic patients had ground-glass opacities (GGO) in their CT chest (*p* value < 0.001) as shown in Table 2 (Fig. 17).

### Vitamin D relationships with inflammatory markers in COVID-19 patients (symptomatic and non-symptomatic)

**Fig. 18:**
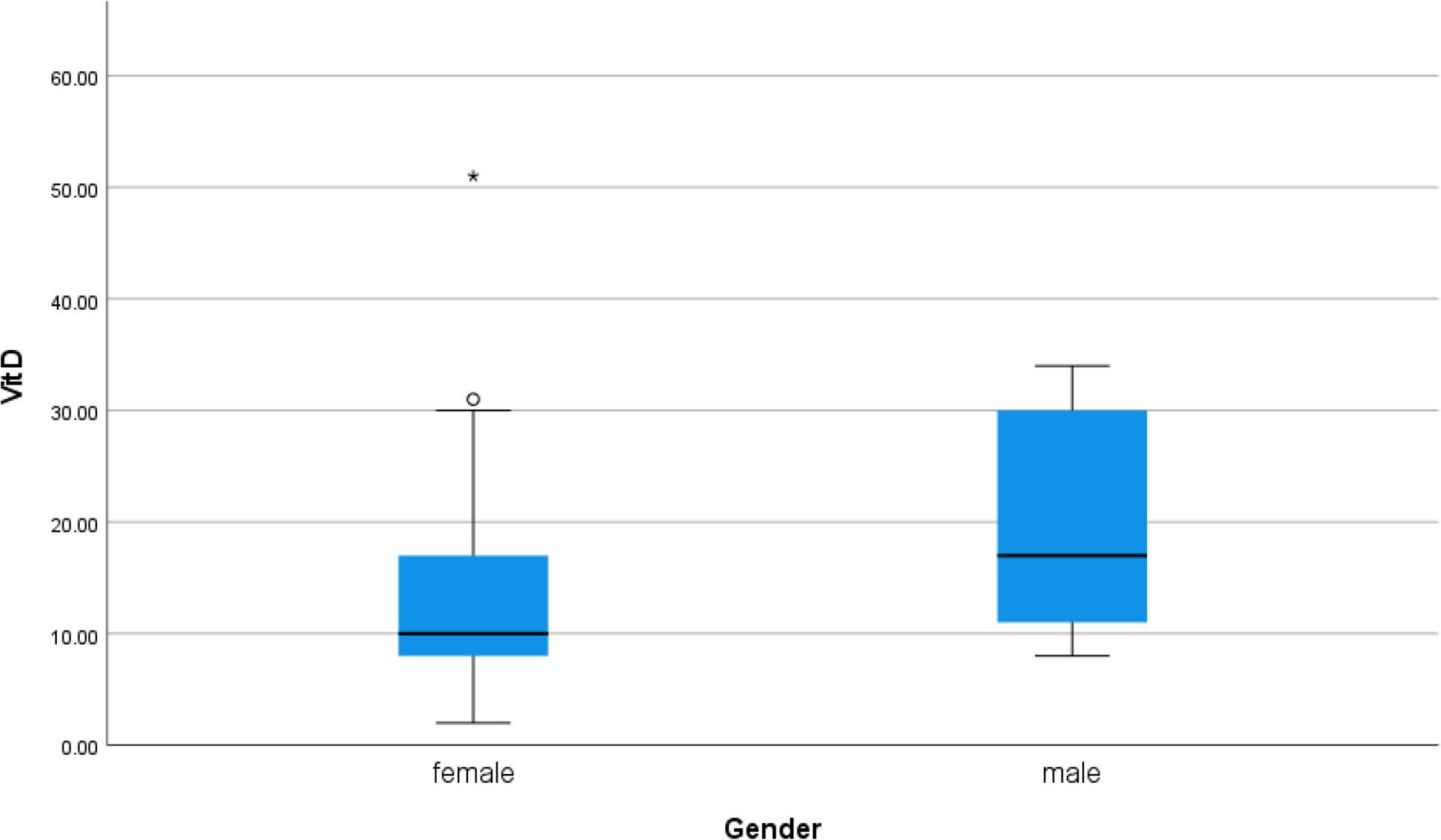
Correlation between serum vitamin D level and sex of COVID 19 patients (symptomatic and non-symptomatic)

Serum vitamin D was low in females than in males (*p* value 0.014) as shown in Table 3 (Fig. 18).

**Table 3:** Relationship between serum vitamin D level and symptoms and comorbidities of the patients

		Vit D					
		Mean	Standard Deviation	Median	Minimum	Maximum	***p*** value
**Gender**	**Male**	19.21	9.77	17.00	8.00	34.00	0.014
	**Female**	13.37	8.63	10.00	2.00	51.00	
**Smoking**	**Yes**	22.00	11.80	26.50	5.00	30.00	0.279
	**No**	14.51	9.01	11.00	2.00	51.00	
**DM**	**Yes**	13.57	9.13	11.00	5.00	28.00	0.505
	**No**	15.08	9.32	12.00	2.00	51.00	
**Obesity**	**Yes**	8.88	3.18	8.50	5.00	15.00	0.026
	**No**	15.70	9.49	13.00	2.00	51.00	
**HTN**	**Yes**	15.25	10.44	11.00	5.00	31.00	0.841
	**No**	14.89	9.18	12.00	2.00	51.00	
**Fever**	**Yes**	14.35	9.40	10.50	4.00	51.00	0.196
	**No**	16.13	9.01	13.00	2.00	34.00	
**Cough**	**Yes**	16.16	10.22	13.00	2.00	51.00	0.233
	**No**	12.93	7.14	9.00	4.00	32.00	
**Anosmia**	**Yes**	13.47	7.98	10.50	2.00	31.00	0.280
	**No**	16.00	10.04	12.00	4.00	51.00	
**Diarrhea**	**Yes**	16.43	10.02	13.00	5.00	32.00	0.643
	**No**	14.56	9.11	11.00	2.00	51.00	
**Severity**	**Moderate risk**	7.33	0.58	7.00	7.00	8.00	0.027
	**Low risk**	15.26	9.31	12.00	2.00	51.00

**Fig. 19:**
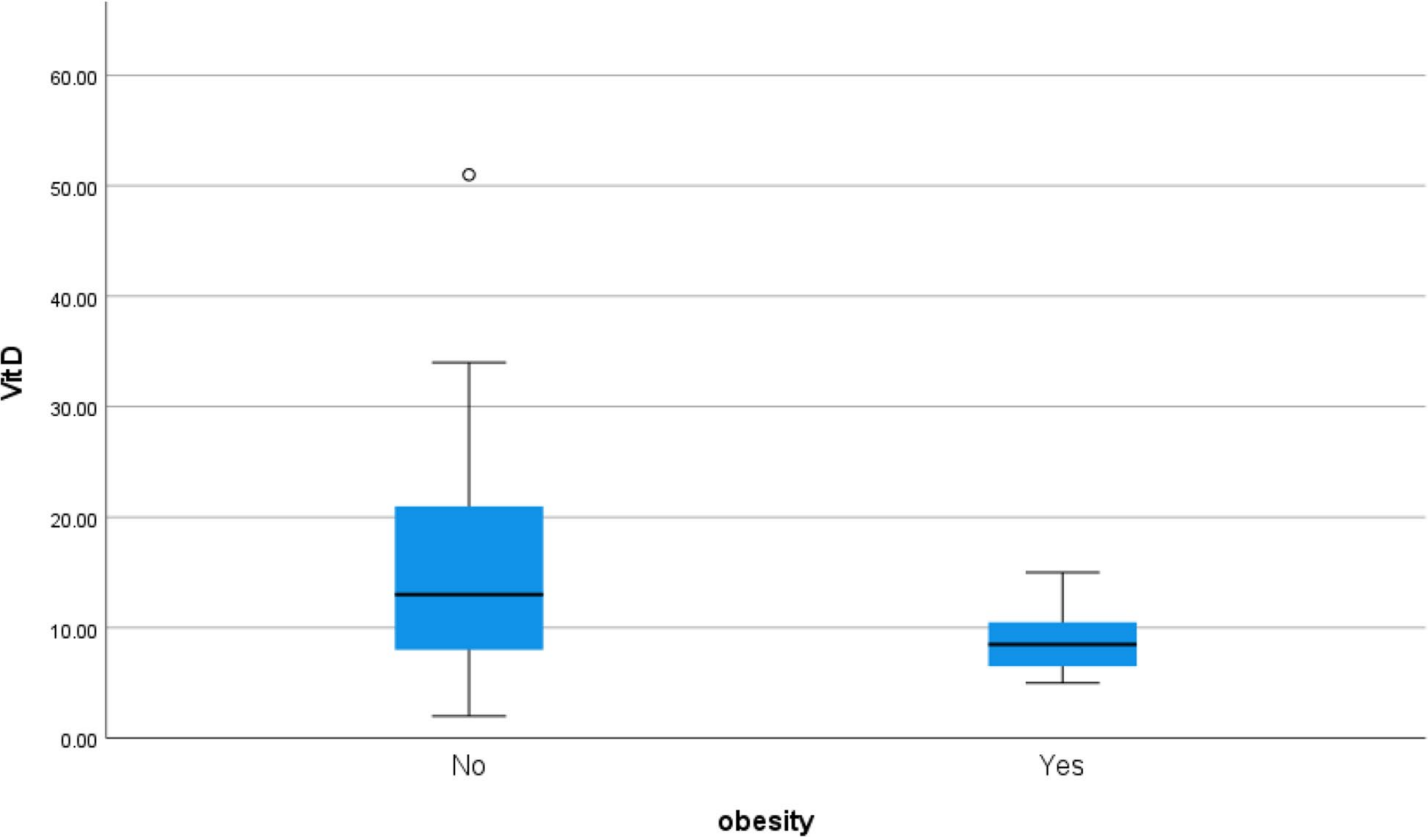
Correlation between serum vitamin D level and obesity in COVID 19 patients (symptomatic and non-symptomatic)

Serum vitamin D was low in obese symptomatic patients (*p* value 0.026) as shown in Table 3 (Fig. 19).

**Fig. 20:**
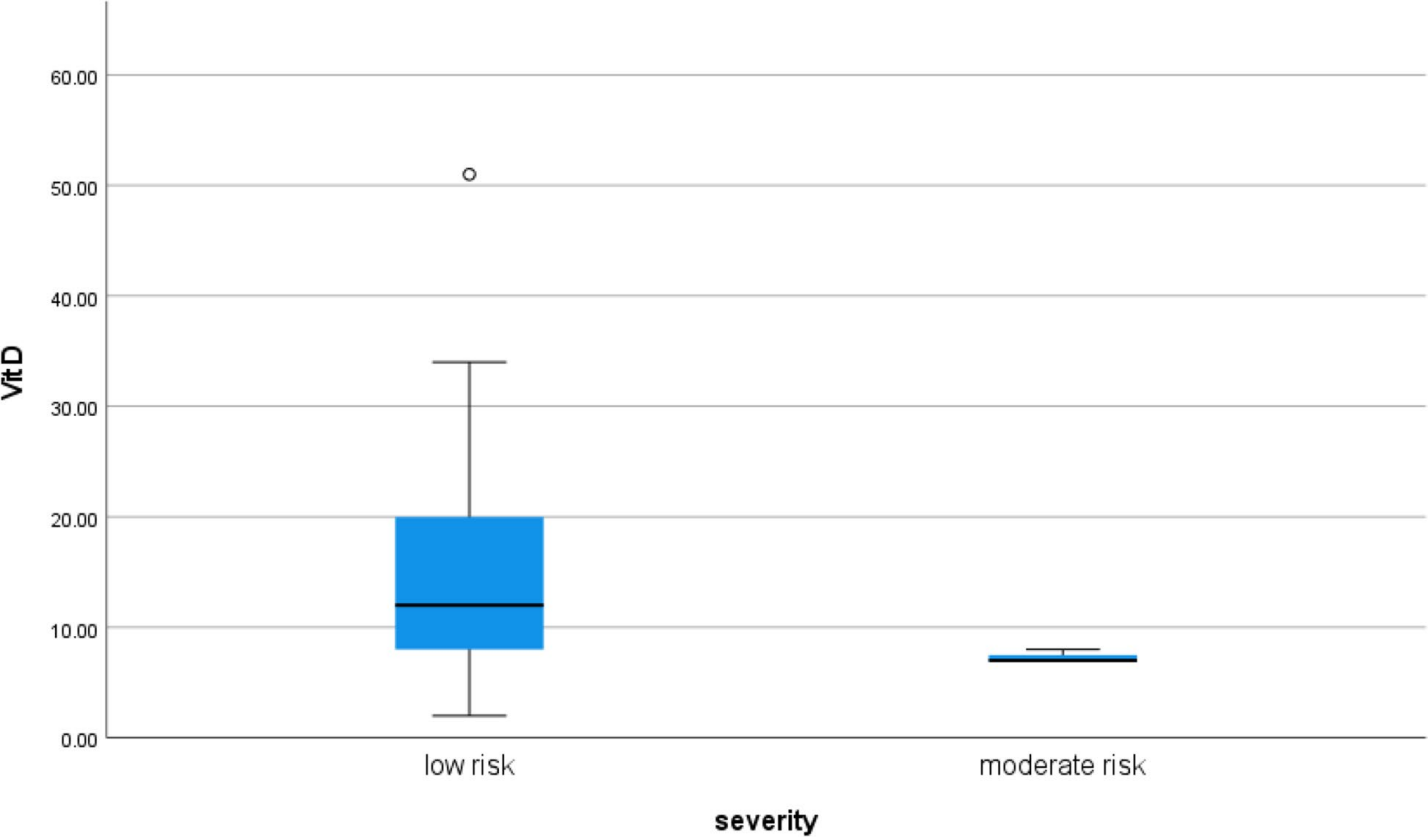
Correlation between serum vitamin D level and the severity of symptoms in COVID 19 patients (symptomatic and non-symptomatic)

Serum vitamin D level was lower in moderate risky symptomatic patients than in mild risky symptomatic patients (*p* value 0.027) as shown in Table 3 (Fig. 20).

Serum ferritin level was high in symptomatic patients with low vitamin D level (*p* value 0.044) as shown in Table 4.

**Table 4:** Correlation between vitamin D and inflammatory markers

	Vit D		
	Correlation coefficient	***p*** value	***N***
**Age**	0.114	0.345	71
**SO2 %**	0.076	0.530	71
**Hb**	0.367	0.002	71
**TLC**	−0.071	0.554	71
**Lymph**	−0.235	0.048	71
**Neutro**	−0.079	0.510	71
**PLT**	−0.046	0.706	71
**Ferritin**	0.240	0.044	71
**CRP**	0.020	0.0271	71
**D-dimer**	0.197	0.039	71
**CT (CORADS)**	0.058	0.633	71

**Fig. 21:**
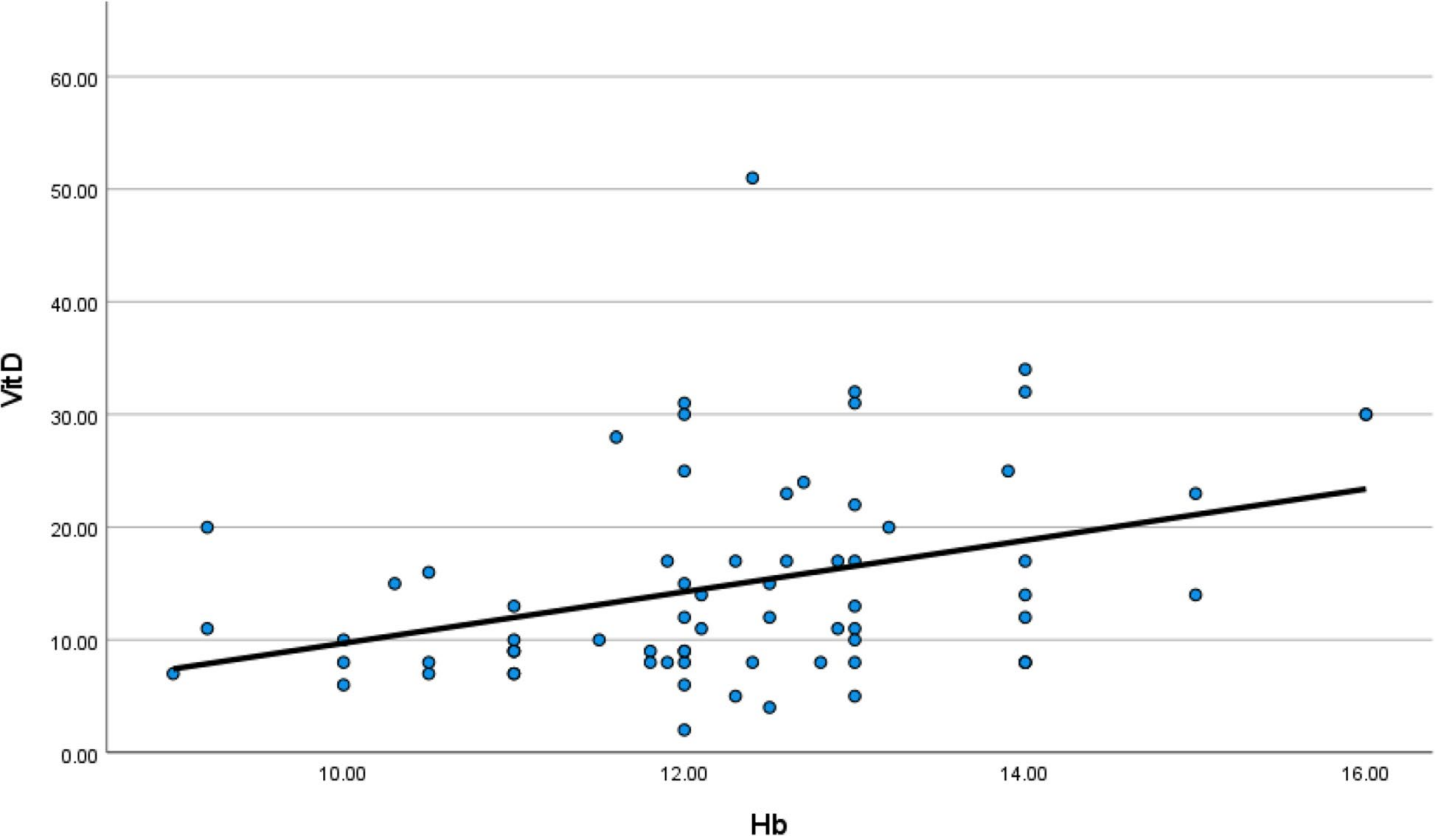
Correlation between serum vitamin D and hemoglobin level in COVID 19 patients (symptomatic and non-symptomatic)

Hemoglobin level and lymphocytes were low in symptomatic patients with low vitamin D level (*p* value 0.002 and 0.048), respectively, as shown in Table 4 (Fig. 21).

**Fig. 22:**
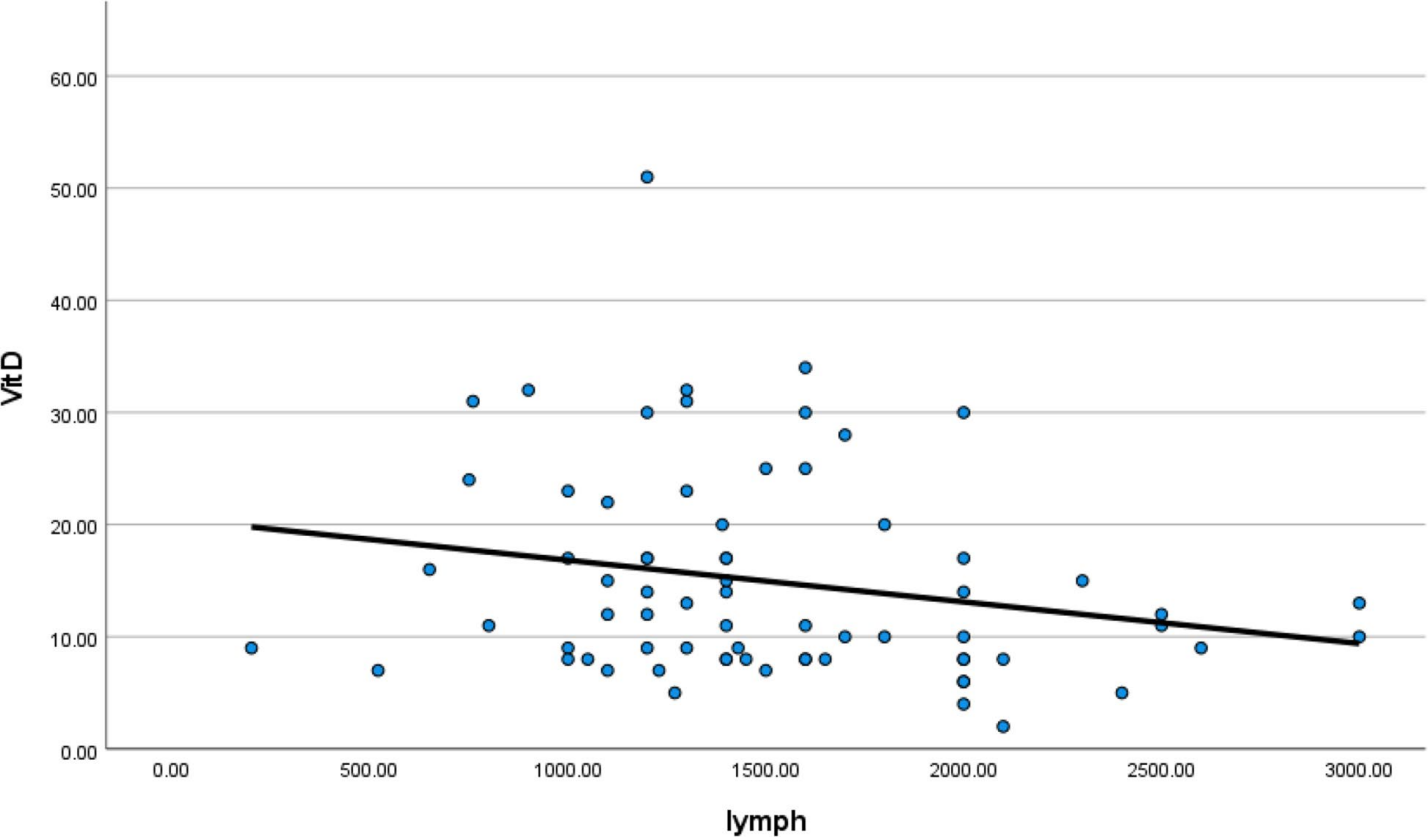
Correlation between serum vitamin D and lymphocytic count in COVID 19 patients (symptomatic and non-symptomatic)

**Fig. 23:**
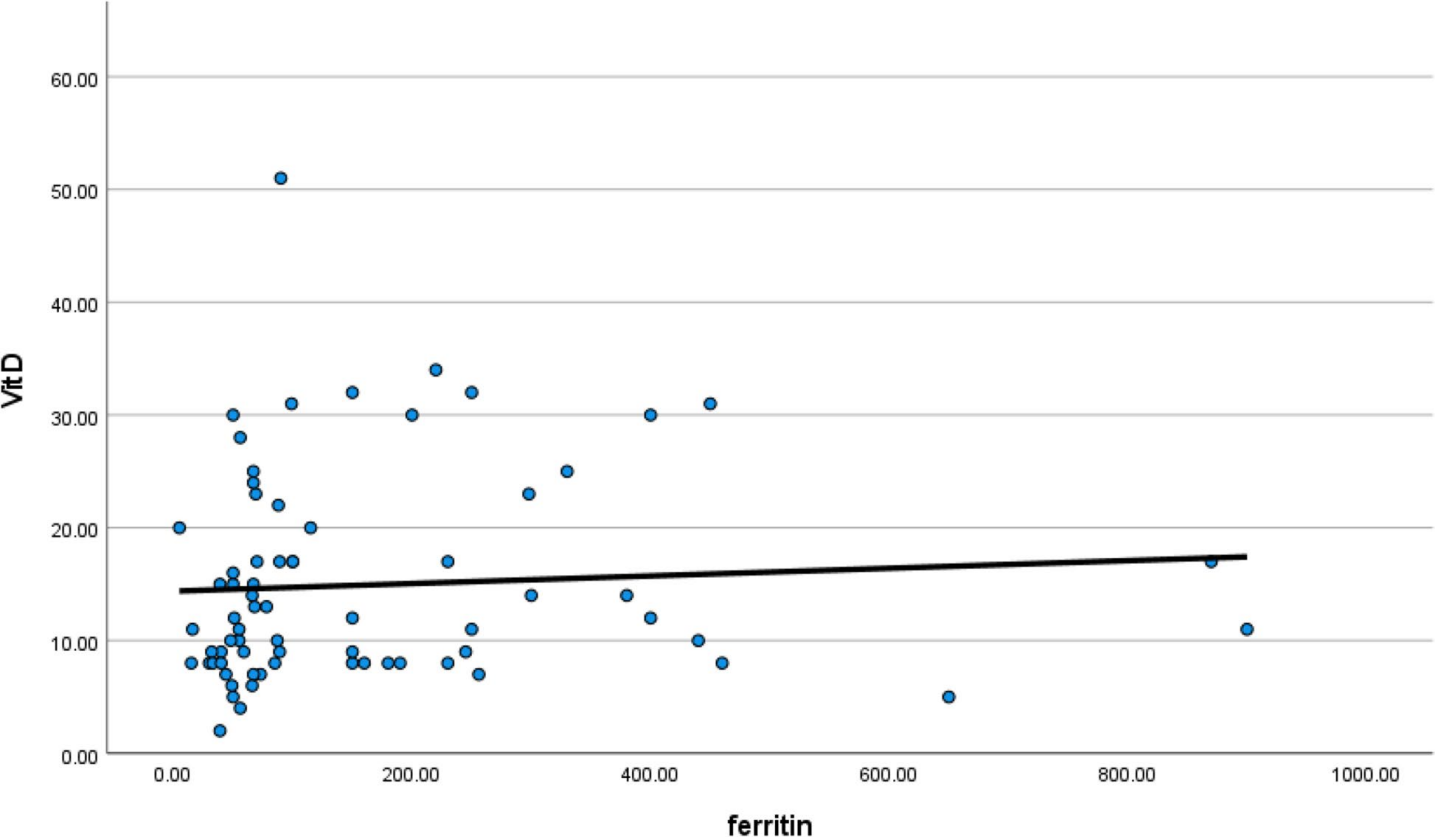
Correlation between serum vitamin D and serum ferritin level in COVID 19 patients (symptomatic and non-symptomatic)

**Fig. 24:**
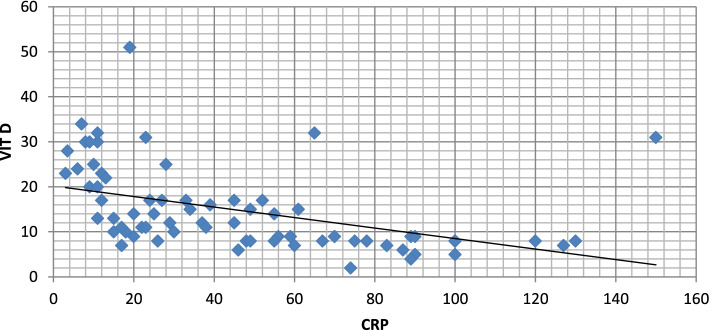
Correlation between vitamin D and CRP level

**Fig. 25:**
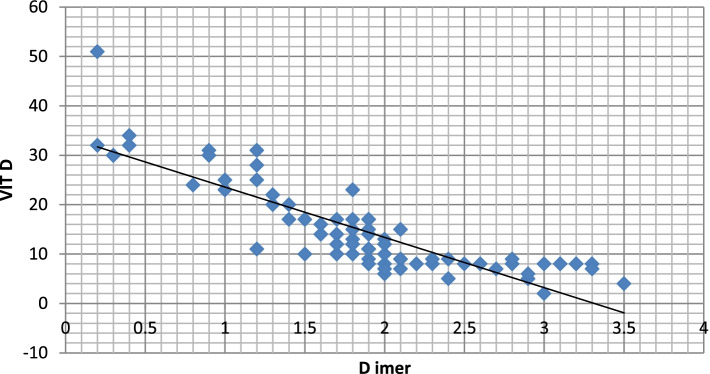
Correlation between vitamin D and D-dimer level

CRP and D-dimer levels were high in symptomatic patients who had low vitamin D level (*p* value 0.0271 and 0.039), respectively, as shown in Table 4 (Figs. 22, 23, 24, and 25).

### Statistical analysis

Data were analyzed using SPSS statistical software, version 20.0 (SPSS, Chicago, IL, USA). All continuous data are presented as means and standard deviations, while categorical data are presented as numbers and percentages.

The Shapiro–Wilk test was used to analyze the distribution of continuous variables. Student’s *t*-test was used to analyze parametric variables, while the Mann–Whitney *U* test was used to analyze non-parametric variables. A chi-square test was used to compare categorical variables. Multivariate regression analysis was performed to analyze relationships between age, CRP level, D-dimer level, fibrinogen level, and vitamin D level.

A primary regression model was generated using a stepwise procedure and included all potential interaction variables. This model was generated using independent variables achieving a *p* value of 0.10 during bivariate analysis. Then, the best-fit model was generated without interaction variables. For all calculations, a *p* value of less than 0.05 was considered statistically significant.

## Discussion

The outbreak of coronavirus disease 2019 (COVID-19), which is caused by the highly contagious severe acute respiratory syndrome coronavirus 2 (SARS-CoV-2), was announced a pandemic in March 2020 by the World Health Organization. The disease mainly affects the respiratory system and spreads via aerosols released during sneezing and coughing [1, 2].

The main symptoms of COVID-19 are fever, cough, runny nose, nasal congestion, shortness of breath, headache, and myalgia [2, 3]. The disease can be diagnosed on the basis of clinical symptoms, polymerase chain reaction positivity, and the presence of ground-glass opacities on computed tomography (CT) scans [4].

Recent studies have focused on the role of serum inflammatory markers that predict COVID-19, such as lymphocyte counts and C-reactive protein (CRP), homocysteine, and D-dimer levels, and their correlation with serum vitamin D level [5, 6].

The aim of our study is that knowing if serum vitamin D level had a correlation with symptoms, severity, and inflammatory markers (CRP, D-dimer, and ferritin) in COVID-19 patients (symptomatic and asymptomatic).

We found that serum vitamin D level was low in females than in males and was low in obese symptomatic patients.

Serum vitamin D level deficiency affected the symptoms and severity of COVID-19 infection as non-symptomatic patients had normal vitamin D level and symptomatic patients had low vitamin D level.

Serum vitamin D level had a positive correlation with hemoglobin level and lymphocytes.

Serum vitamin D had a negative correlation with serum ferritin, CRP, and D-dimer and was not correlated with CORAD scoring in CT chest.

We are in concur with Anshul Jain et al. (*Sci Rep*. [11]). The aim of their study is to analyze the vitamin D level in COVID-19 patients and its impact on the disease severity. The researchers found that serum level of inflammatory markers was found to be higher in vitamin D-deficient COVID-19 patients. They also found that symptoms of COVID-19 infection were more severe in patients with a low level of serum vitamin D.

Our work is also in concur with Federica Saponaro et al. (*Front Immunol*. [12]). The aim of this study is to analyze the relationship between vitamin D status and a biochemical panel of inflammatory markers in a cohort of patients with COVID-19. The results showed that a significant inverse correlation was found between 25OHD and all inflammatory markers (serum ferritin, CRP, and D-dimer), even adjusted for age and sex.

Our study is in concur with Mazen Almehmadi1 et al. [13]*.* Their results showed that vitamin D levels were inversely correlated with the markers used for monitoring the condition of COVID-19 patients: ferritin, CRP, and D-dimer, and serum vitamin D was low in symptomatic patients and normal in non-symptomatic patients.

Our work is in contrast to Ola Alsegai et al. [14]. The researchers did not observe any significant differences in the serum 25(OH)D levels among our critically ill adults who died and who were alive at the time of their admission, and there were significant differences in serum vitamin D and inflammatory markers (CRP and serum ferritin).

In conclusion, serum vitamin D was low in symptomatic patients and normal in non-symptomatic patients. Serum vitamin D was inversely correlated with inflammatory markers (ferritin, CRP, and D-dimer).

Serum vitamin D level deficiency affected the symptoms and severity of COVID-19 infection.

So vitamin D level deficiency affected symptoms, severity, and inflammatory markers.

Vitamin D level may be used as a predictor for the severity of COVID-19 infection.

## Abbreviations

CRP: C-reactive protein
CT: Computed tomography
GGO: Ground-glass opacities
HPLC: High-performance liquid chromatography
NEWS: National Early Warning Score
PCR: Polymerase chain reaction
RIA: Radioimmunoassay
